# Association Between Patient Portal Engagement and Weight Loss Outcomes in Patients After Bariatric Surgery: Longitudinal Observational Study Using Electronic Health Records

**DOI:** 10.2196/56573

**Published:** 2024-12-09

**Authors:** Xinmeng Zhang, Kaidi Kang, Chao Yan, Yubo Feng, Simon Vandekar, Danxia Yu, S Trent Rosenbloom, Jason Samuels, Gitanjali Srivastava, Brandon Williams, Vance L Albaugh, Wayne J English, Charles R Flynn, You Chen

**Affiliations:** 1 Department of Computer Science Vanderbilt University Nashville, TN United States; 2 Department of Biostatistics Vanderbilt University Medical Center Nashville, TN United States; 3 Department of Biomedical Informatics Vanderbilt University Medical Center Nashville, TN United States; 4 Division of Epidemiology Department of Medicine Vanderbilt University Medical Center Nashville, TN United States; 5 Department of Pediatrics Vanderbilt University School of Medicine Nashville, TN United States; 6 Department of Surgery Vanderbilt University School of Medicine Nashville, TN United States; 7 Division of Diabetes, Endocrinology & Metabolism Department of Medicine Vanderbilt University School of Medicine Nashville, TN United States; 8 Vanderbilt Weight Loss Center Vanderbilt University Medical Center Nashville, TN United States; 9 Metamor Institute Pennington Biomedical Research Center Baton Rouge, LA United States

**Keywords:** patient portal, patient engagement, bariatric surgery, retrospective, longitudinal study, data-driven, weight loss, obesity, electronic health records, postoperative care

## Abstract

**Background:**

Bariatric surgery is an effective intervention for obesity, but comprehensive postoperative self-management is essential for optimal outcomes. While patient portals are generally seen as beneficial in engaging patients in health management, the link between their use and post–bariatric surgery weight loss remains unclear.

**Objective:**

This study aimed to investigate the association between patient portal engagement and postoperative BMI reduction among patients after bariatric surgery.

**Methods:**

This retrospective longitudinal study included patients who underwent Roux-en-Y gastric bypass or sleeve gastrectomy at Vanderbilt University Medical Center between January 2018 and March 2021. Patient portal engagement was measured during 4 stages: early (within 3 months after surgery), early midterm (3-6 months), late midterm (6-9 months), and late (9-12 months). Using generalized estimating equations, we estimated the associations between patients’ portal engagements at these stages and the percentage of BMI reduction (%BMIR) at 3, 6, and 12 months after surgery. Covariates included duration since surgery, patient’s age at the time of surgery, sex, race and ethnicity, type of bariatric surgery, severity of comorbid conditions, and socioeconomic disadvantage.

**Results:**

The study included 1415 patients, predominantly female (n=1145, 80.9%), with a racial composition of 76.9% (n=1088) White and 19.9% (n=282) Black. Overall, 805 (56.9%) patients underwent Roux-en-Y gastric bypass and 610 (43.1%) underwent sleeve gastrectomy. By 1 year after surgery, the median %BMIR was 31.5% (IQR 25.2%-36.8%), and the median number of active days on the patient portal was 54 (IQR 33-80). Early portal engagement was significantly associated with %BMIR at various postoperative times. Specifically, each additional 10 days of early portal engagement was associated with a 0.37% (95% CI –0.55% to –0.18%; *P*<.001) lower expected %BMIR at 3 months, a 1.11% (95% CI 0.82%-1.41%; *P*<.001) higher expected %BMIR at 6 months, and a 0.78% (95% CI 0.25%-1.31%; *P*=.004) higher expected %BMIR at 12 months. Furthermore, early midterm portal engagement was associated with a 0.36% (95% CI –0.69 to –0.03; *P*=.03) lower expected %BMIR at 6 months, but it was not significant at 12 months (*P*=.88). Late midterm and late portal engagement were not significantly associated with %BMIR at 12 months (*P*=.27 and *P*=.12, respectively). Furthermore, early engagement in various portal functions, such as messaging and accessing medical records, was significantly associated with a lower %BMIR at 3 months and a higher %BMIR at both 6 and 12 months (all *P*<.05).

**Conclusions:**

Higher patient portal engagement within 3 months after surgery—suggestive of stronger adherence to postoperative instructions and improved communication with care teams—is associated with less favorable weight loss immediately after surgery but enhanced postoperative weight loss outcomes at 6 and 12 months. However, the limitations of retrospective data-driven studies highlight the need for future intervention-based studies to validate these associations and establish causality.

## Introduction

Obesity continues to be a significant health concern in the United States, with approximately 42% and 9.2% of adults classified as having obesity (BMI≥30 kg/m^2^) and severe obesity (BMI≥40 kg/m^2^), respectively [1]. Research has consistently demonstrated that individuals with obesity face an elevated risk for a range of health complications, including, but not limited to, cardiovascular disease, diabetes, respiratory problems, and metabolic syndrome [2-6]. Bariatric surgery remains the most effective intervention for individuals who struggle to achieve sustainable weight loss solely through diet and exercise, whether through its effects of restricting food intake or modifying the digestive system [7,8].

Comprehensive postoperative care following hospital discharge plays a pivotal role in achieving optimal outcomes and ensuring success for patients after bariatric surgery [9]. Effective health tracking, patient self-management, and efficient collaboration between patients and their care teams are of utmost importance in working toward shared goals, including sustained weight loss, improved health conditions, and enhanced overall quality of life. Such postoperative needs have been increasingly supported by both web-based and mobile app–based patient portals [10], which typically provide patients access to their own electronic health information and serve as platforms for timely communication and regular health information exchange with their care teams. Growing evidence underscores the efficacy of portal usage in delivering continuous support and guidance, monitoring key indicators related to primary interventions, enhancing medication adherence, and fostering self-management in promoting behavioral and lifestyle modifications [11-16]. Although the multifaceted benefits of patient portals in health care settings are well documented, the specific impact of portal use on BMI changes in patients after bariatric surgery remains underexplored. Understanding the time-varying association between portal engagement at various critical stages (ie, early, midterm, and late) and the percentage of BMI reduction (%BMIR) is crucial, as it can provide valuable insights into the optimal timing and types of portal interactions that most effectively support postsurgery weight management. By identifying the most influential timing for portal engagement, health care providers can tailor their digital interventions to maximize their impact, thus enhancing patient adherence, self-management, and ultimately, health outcomes.

In this study, we investigated the association between patient portal engagement at various stages—early (within 3 months after surgery), early midterm (3 to 6 months), late midterm (6 to 9 months), and late (9 to 12 months)—and the %BMIR at 3, 6, and 12 months after surgery. We hypothesized that portal engagement at these different stages has varying associations with %BMIR at various postoperative times. Should the hypothesis test results reveal significant differences in the association across these stages, such findings would prompt a re-evaluation of current postoperative care practices.

## Methods

### Ethical Considerations

This study was approved by the institutional review boards (IRBs) at Vanderbilt University Medical Center (VUMC) under IRB#221459. A full waiver of written informed consent from patients was granted by the IRB because this study is retrospective with minimal risks to patients and the study data are deidentified. This study follows the STROBE (Strengthening the Reporting of Observational Studies in Epidemiology) reporting guideline.

### Study Settings

We conducted a single-site, retrospective, longitudinal study at VUMC, a large academic medical center in Nashville, Tennessee, that provides primary and specialty referral care to patients from across the Southeastern United States. This study included patients who underwent Roux-en-Y gastric bypass (RYGB) or sleeve gastrectomy (SG) operations, the most commonly performed operations [17], between January 1, 2018, and March 1, 2021, inclusive, as part of their participation in the VUMC Surgical Weight Loss Program. The specific end date reflected the most current data update within the available dataset. The VUMC Surgical Weight Loss Program is accredited by the Metabolic and Bariatric Surgery Accreditation and Quality Improvement Program (MBSAQIP) [18]. VUMC deployed its patient portal, My Health at Vanderbilt (MHAV), in 2004, and migrated to Epic’s MyChart platform in late 2017 as part of a wider electronic health record (EHR) system update across VUMC. Like most patient portals, MHAV allows patients to access their own electronic health information, make appointments, manage medications, and interact with their care providers through a secure messaging system [19]. MHAV currently has over 1 million users and is accessed over 30 million times annually.

### Data

The cohort was obtained from the VUMC bariatric surgery Quality, Efficacy, and Safety registry, a VUMC-specific database used exclusively for internal research and quality enhancement. We considered initial RYGB or SG operations performed at VUMC and excluded any subsequent surgical revisions due to their inherent complexity. We identified the date of each patient’s initial bariatric surgery (day 0) as the index event. Following the surgery, patients were scheduled for clinic follow-ups at 3, 6, and 12 months after surgery. We collected BMI data from the Quality, Efficacy, and Safety registry and EHR. The observations with missing weights at the follow-up visits at 3, 6, or 12 months after surgery and the patients who lacked weight records immediately before surgery (baseline) were excluded.

Patient demographic and clinical information were extracted from the Epic EHR system, which was deployed at VUMC in 2017. Demographic information includes age at surgery, sex (female or male), and self-reported race and ethnicity (Black, White, or other races or ethnicities) from the EHR. We grouped American Indian or Alaska Native, Asian, Asian Indian, Chinese, Cuban, Filipino, Guamanian or Chamorro, Hispanic or Latino, Japanese, Korean, Mexican, Mexican American or Chicano, Native Hawaiian, other Asian, other Pacific Islander, Puerto Rican, Samoan, Vietnamese, and none of the above into “other races or ethnicities” to avoid unstable estimates due to their small cohort sizes. Past medical conditions of each patient, encompassing a period of 10 years up to the index event, were extracted to calculate the Charlson Comorbidity Index (CCI) [20,21]. To account for socioeconomic status, we used the Area Deprivation Index (ADI) [22], a widely adopted measure indicating the neighborhood’s socioeconomic disadvantage level. In our analysis, we determined patients’ ADI by their respective 5-digit zip codes. All patients did not have missing data on the type of surgery, the time of surgery, sex, age, and zip code. We excluded patients who had missing records of race and ethnicity. Study cohort inclusion and exclusion criteria were detailed in Figure S1 in Multimedia Appendix 1.

We extracted patient portal engagement history from MHAV event logs, which record every action taken by system users through provided interfaces [23-28]. These included unique patient identifiers, event time stamps, and event types. For each patient, we focused on the time frame starting from day 5 after surgery—typically regarded as the commencement of the postdischarge phase—up to 12 months after surgery. We categorized event types into 7 portal functions based on previous literature, which are messaging (support communication between health care providers and patients), visits (facilitate appointment management), my record (provide lists of allergies, immunizations, medical history, medications, pharmacy, preventive care, test results, and vitals), medical tools (allow patients to view document and add devices), billing (support account payment and insurance management), resources (provide patient education materials), and others (include additional functions not specified above, such as “Send proxy invite”) [23]. The full list of event types associated with these functions is reported in Table S1 in Multimedia Appendix 1.

### Measure Definition

There is no universally agreed-upon measure of patient portal engagement. In this study, we quantified engagement by measuring the number of days in which a patient performed any action within MHAV after logging in, as recorded in the event logs of MHAV. Days that solely consisted of log-in and log-out events were not counted. As such, we use “active days of portal use” interchangeably to refer to this measurement. We tracked the number of active portal usage days at various postoperative stages to evaluate the effects of portal engagement at different stages over time. Specifically, we categorized the engagement as follows: “early engagement” from day 5 to 3 months after surgery, “early midterm engagement” from 3 to 6 months, “late midterm engagement” from 6 to 9 months, and “late engagement” from 9 to 12 months.

Postoperative weight loss outcome is defined as the %BMIR at month *m* compared with the baseline BMI (kg/m^2^):

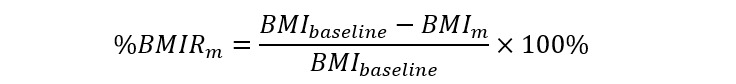



We selected %BMIR because it accounts for differences in baseline body weight and provides standardized comparisons across diverse patient populations. Compared with other weight loss measures, such as percentage of weight loss and percentage of excess weight loss, %BMIR provides a more sensitive and consistent indicator of surgery-induced weight loss [29,30]. The primary goal of this study is to examine the time-varying association between patients’ portal engagements at different postoperative stages and %BMIR.

### Statistical Analysis

The data analysis was performed between February 2023 and November 2023. A descriptive analysis was performed to summarize the demographic and clinical characteristics of the bariatric surgery cohort. We used generalized estimating equations [31] with an identity link function and an autoregressive correlation structure (ie, AR-1) for statistical modeling. The use of the AR-1 correlation structure allows for the consideration of the time-dependent nature of within-patient correlation in %BMIR, ensuring the closer sequential measurements are more strongly correlated. The independent and dependent variables are the number of active days of portal use at different stages (winsorized at 95th percentile to limit extreme values and reduce the influence of outliers) and the %BMIR, respectively. All independent variables were included in the same model. We included the following covariates in all the models to control possible confounding: the duration since surgery (in months), the patient’s age at the time of surgery (in years), sex (female or male), race and ethnicity (Black, White, or other races or ethnicities), type of bariatric surgery (RYGB or SG), CCI score, and ADI. The covariates were chosen based on previous literature, which was detailed in the conceptual framework (Figure S2 in Multimedia Appendix 1). Furthermore, to model the time-varying effects of patient portal engagement amount at different postoperative stages on the %BMIR, we included the interaction terms between portal engagement at different stages and the duration since surgery (in months). In addition to the multivariate analyses, the univariate generalized estimating equation models were also fitted to explore the effects of the early, early midterm, late midterm, and late engagements on the outcome, separately. There were no missing data for the covariates. All analyses used complete cases for %BMIR at the available follow-up time points.

We centered our analyses around the investigation of the association between patient portal engagement at various stages (ie, early, early midterm, late midterm, and late portal engagement) and the %BMIR at various postoperative times (ie, 3, 6, and 12 months after surgery). Our primary hypothesis is that portal engagement at these distinct stages has different associations with %BMIR at these respective time points. We also conducted a secondary analysis to test the relative influence of specific portal functions. As part of this, we restricted our analysis to the active days of each portal function separately. A sensitivity analysis was performed by adding BMI immediately before surgery (BMI at baseline) to the primary model to account for its potential mediation effect. In addition, considering the overlap of our study period with the COVID-19 pandemic, during which MHAV usage spiked as a portion of health care moved to virtual delivery [32], we conducted an additional sensitivity analysis by dividing the patient cohort based on whether their surgeries occurred before March 1, 2020. We then determined if the associations between portal engagement and %BMIR were consistent before and during the pandemic. Considering that older patients may experience different levels of success with digital tools compared with younger adults [33], we conducted a sensitivity analysis to assess the influence of age on the relationship between portal engagement at various postoperative stages and weight loss outcomes. This analysis included interaction terms between stages of portal engagement and patient age.

All statistical analyses were conducted using R software (version 4.3.1; R Core Team). A *P* value less than .05 was considered statistically significant. In addition to *P* values, effect size estimates were also reported using a robust effect size index (RESI) along with their 95% CIs [34,35]. The RESI is equal to 0.5 Cohen's *d* under some assumptions [36], so Cohen's *d*–suggested interpretations for effect size are as follows: none to small (RESI=0-0.1), small to medium (RESI=0.1-0.25), and medium to large (RESI=0.25-0.4). The direction of the RESI estimates indicates the association direction.

## Results

### Descriptive Analysis

The cohort of those who underwent bariatric surgery comprised 1415 patients, with 3377 observations within 1 year of follow-up visits (the study cohort flow diagram is illustrated in Figure S1 in Multimedia Appendix 1). In the included cohort, there were no missing data in the covariates. There were 805 (56.9%) patients who underwent RYGB and 610 (43.1%) who underwent SG. There were 1145 (80.9%) female patients (Table 1). The mean age as of the surgery date was 44.5 (SD 11.4) years, with a median baseline BMI of 45.5 (IQR 41.4-51.4) kg/m^2^. After surgery, 95.1% (n=1345), 76.7% (n=1086), and 66.9% (n=946) of patients had a follow-up at the 3, 6, and 12 months, respectively. The median %BMIR at the 3, 6, and 12 months was 15.8% (IQR 13.7%-18.1%), 24.4% (IQR 20.4%-27.9%), and 31.5% (IQR 25.2%-36.8%), respectively. Over 98% of patients who underwent bariatric surgery used MHAV at least once after surgery. Patients had a median engagement of 23 (IQR 15-33), 35 (IQR 21-50), and 54 (IQR 33-80) active days within the first 3, 6, and 12 months after surgery, respectively.

**Table 1:** Characteristics of study cohort.

Characteristics			Overall (N=1415)		Portal active days Q1^a,b^ (n=349)		Portal active days Q2^a,c^ (n=352)	Portal active days Q3^a,d^ (n=354)		Portal active days Q4^a,e^ (n=360)
**BMI records missing, n (%)**										
	3-month follow-up	70 (4.9)		19 (5.4)		14 (4)		17 (4.8)	20 (5.6)	
	6-month follow-up	329 (23.3)		126 (36.1)		92 (26.1)		74 (20.9)	37 (10.3)	
	12-month follow-up	469 (33.1)		201 (57.6)		131 (37.2)		87 (24.6)	50 (13.9)	
**Sex, n (%)**										
	Female	1145 (80.9)		266 (76.2)		285 (81)		305 (86.2)	289 (80.3)	
	Male	270 (19.1)		83 (23.8)		67 (19)		49 (13.8)	71 (19.7)	
**Race and ethnicity, n (%)**										
	Black	282 (19.9)		91 (26.1)		66 (18.8)		61 (17.2)	64 (17.8)	
	White	1088 (76.9)		246 (70.5)		278 (79)		285 (80.5)	279 (77.5)	
	Other races or ethnicities	45 (3.2)		12 (3.4)		8 (2.3)		8 (2.3)	17 (4.7)	
**Operation, n (%)**										
	RYGB^f^	805 (56.9)		187 (53.6)		193 (54.8)		214 (60.5)	211 (58.6)	
	SG^g^	610 (43.1)		162 (46.4)		159 (45.2)		140 (39.5)	149 (41.4)	
**Age at baseline (years), mean (SD)**			44.5 (11.4)		45.9 (11.6)		43.6 (11.5)	43.7 (10.7)		44.8 (11.7)
**Area Deprivation Index, mean (SD)**			56.3 (19.1)		59.4 (18.8)		56.5 (18.9)	57.0 (18.9)		52.3 (19.2)
**Charlson Comorbidity Index, mean (SD)**			1.23 (1.59)		1.13 (1.43)		0.963 (1.41)	1.21 (1.60)		1.62 (1.79)
**Baseline BMI (kg/m^2^), median (IQR)**			45.5 (41.4-51.4)		45.5 (41.1-51.0)		44.8 (41.3-50.7)	45.7 (41.4-51.1)		45.6 (41.6-52.6)
**%BMIR, median (IQR)**										
	3 months	15.8 (13.7-18.1)		15.6 (13.4-17.8)		15.8 (13.8-18.3)		15.9 (13.6-18.1)	16.0 (14.0-18.1)	
	6 months	24.4 (20.4-27.9)		23.1 (19.8-27.4)		25.1 (21.1-28.4)		24.1 (20.4-27.7)	24.4 (20.8-28.2)	
	12 months	31.5 (25.2-36.8)		30.8 (24.7-35.6)		32.7 (26.8-37.4)		31.4 (25.2-36.1)	31.5 (25.1-37.3)	
**Portal active days^h^, median (IQR)**										
	Early engagement	23.0 (15.0-33.0)		10.0 (4.0-15.0)		20.0 (16.0-25.0)		27.0 (22.0-32.0)	38.0 (32.0-45.0)	
	Early midterm engagement	11.0 (5.0-18.0)		3.0 (1.0-6.0)		8.0 (5.0-11.0)		14.0 (11.0-17.0)	25.0 (19.0-34.0)	
	Late midterm engagement	10.0 (4.0-17.0)		2.0 (0-5.0)		7.0 (4.75-10.0)		12.0 (9.0-16.0)	22.0 (17.0-30.0)	
	Late engagement	8.0 (3.0-15.0)		1.0 (0-3.0)		5.0 (3.0-9.0)		11.0 (7.0-15.0)	21.0 (14.0-31.0)	
**Function active days at 12 months^a^, median (IQR)**										
	Billing	16.0 (7.0-27.0)		5.0 (1.0-10.0)		13.0 (7.0-20.0)		21.5 (14.0-30.0)	32.0 (21.0-45.0)	
	Medical tools	26.0 (4.0-61.0)		2.0 (0-9.0)		28.0 (4.0-42.0)		48.0 (9.25-62.0)	90.0 (42.5-108)	
	Messaging	32.0 (20.0-50.0)		12.0 (6.0-17.0)		27.0 (22.8-31.0)		40.0 (33.0-48.0)	61.0 (50.0-77.0)	
	My record	52.0 (32.0-78.0)		19.0 (9.0-26.0)		41.0 (37.0-47.0)		63.0 (57.0-70.0)	102 (89.0-124.0)	
	Resources	0 (0-10.0)		0 (0-0)		0 (0-2.0)		0 (0-17.8)	9.0 (0-38.0)	
	Visit	49.0 (27.0-75.0)		16.0 (8.0-24.0)		38.0 (33.0-45.0)		61.0 (55.0-68.8)	97.0 (85.0-122)	
	Others	9.0 (4.0-16.0)		2.0 (1.0-5.0)		7.0 (4.0-9.25)		12.0 (8.0-16.0)	21.0 (13.0-32.3)	

^a^Patient characteristics were stratified by quartiles of portal active days at 12 months. The overall median of portal active days at 12 months was 54 (IQR 33-80). Quartile 1 included patients with fewer than 33 portal active days, quartile 2 included patients with 33 to 54 portal active days, quartile 3 included patients with 54 to 80 portal active days, and quartile 4 included those with more than 80 portal active days.

^b^Q1: quartile 1.

^c^Q2: quartile 2.

^d^Q3: quartile 3.

^e^Q4: quartile 4.

^f^RYGB: Roux-en-Y gastric bypass.

^g^SG: sleeve gastrectomy.

^h^The portal active days were not winsorized.

### Multivariate Analysis

**Figure 1:**
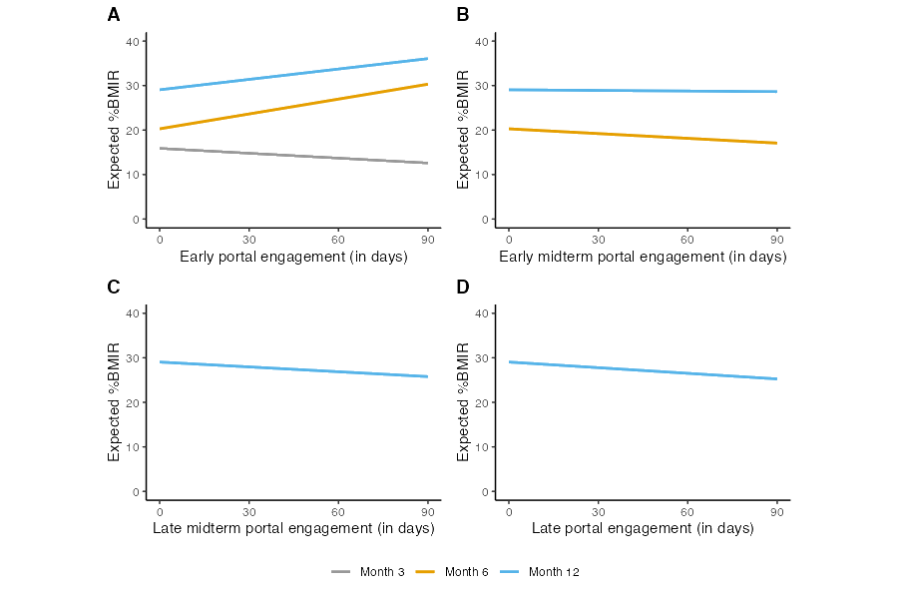
The expected percentage of BMI reduction over patient portal engagement at different stages by follow-up time, after fixing other covariates at the reference levels (ie, age set at 45 years, female sex, race and ethnicity of White, Charlson Comorbidity Index of 0, and Area Deprivation Index of 0). (A) Early portal engagement, (B) early midterm portal engagement, (C) late midterm portal engagement, and (D) late portal engagement.

Our results revealed significant associations between patient early portal engagement and %BMIR: an additional 10-day increment in early portal engagement was associated with a 0.37% (95% CI –0.55% to –0.18%; *P*<.001) lower expected %BMIR at the 3-month follow-up (Table 2). At 6-month and 12-month follow-ups, an additional 10-day increment in early portal engagement was associated with a 1.11% (95% CI 0.82%-1.41%; *P*<.001) and 0.78% (95% CI 0.25%-1.31%; *P*=.004) higher expected %BMIR, respectively (Table 2). Early midterm portal engagement was associated with a 0.36% (95% CI –0.69% to –0.03%; *P*=.03) lower expected %BMIR at the 6-month follow-up. Its association with the %BMIR at the 12-month follow-up was not significant (β=–.05, 95% CI –0.70 to 0.61; *P*=.88). Late midterm portal engagement (β=–.36, 95% CI –1.01 to 0.28; *P*=.27) or late portal engagement (β=–.42, 95% CI –0.96 to 0.12; *P*=.12) did not show significant associations with the %BMIR at 12 months. Figure 1 visualizes the relationship between patient portal engagement at different time stages and expected %BMIR over follow-up time. Univariate analyses showed coherent average marginal effects of portal engagements at different time stages over follow-up time, respectively (Table S2 in Multimedia Appendix 1).

**Table 2:** Model results of the relationship between portal engagement and percentage of BMI reduction over time following bariatric surgery. All other independent variables were factored in as covariates.

Factor	Regression coefficient (95% CI)	*P* value	RESI^a^ (95% CI)
Intercept	16.98 (15.41 to 18.55)	<.001	0.56 (0.50 to 0.64)
Early engagement at 3-month follow-up (per 10-day)^b^	–0.37 (–0.55 to –0.18)	<.001	–0.10 (–0.15 to –0.05)
Early engagement at 6-month follow-up (per 10-day)^b^	1.11 (0.82 to 1.41)	<.001	0.20 (0.14 to 0.26)
Early engagement at 12-month follow-up (per 10-day)^b^	0.78 (0.25 to 1.31)	.004	0.08 (0.02 to 0.13)
Early midterm engagement at 6-month follow-up (per 10-day)^b^	–0.36 (–0.69 to –0.03)	.03	–0.06 (–0.11 to –0.00)
Early midterm engagement at 12-month follow-up (per 10-day)^b^	–0.05 (–0.70 to 0.61)	.88	–0.00 (–0.06 to 0.05)
Late midterm engagement at 12-month follow-up (per 10-day)^b^	–0.36 (–1.01 to 0.28)	.27	–0.03 (–0.08 to 0.02)
Late engagement at 12-month follow-up (per 10-day)^b^	–0.42 (–0.96 to 0.12)	.12	–0.04 (–0.09 to 0.01)
Age (per 5-years)	–0.28 (–0.39 to –0.16)	<.001	–0.12 (–0.18 to –0.07)
Male^c^	0.19 (–0.43 to 0.81)	.55	0.02 (–0.04 to 0.06)
Black^d^	–2.64 (–3.28 to –1.99)	<.001	–0.21 (–0.26 to –0.16)
Other races or ethnicities^d^	–1.55 (–3.24 to 0.14)	.07	–0.05 (–0.11 to 0.00)
ADI^e^	0.00 (–0.01 to 0.02)	.59	0.01 (–0.04 to 0.07)
CCI^f^	–0.30 (–0.47 to –0.13)	<.001	–0.09 (–0.15 to –0.04)
SG^g,h^	–2.97 (–3.48 to –2.47)	<.001	–0.31 (–0.36 to –0.25)
Months post operation	1.46 (1.35 to 1.58)	<.001	0.66 (0.59 to 0.73)

^a^RESI: robust effect size index.

^b^The variables are interaction terms between portal engagement at different stages and the duration since surgery.

^c^The reference group for sex is female.

^d^The reference group for race and ethnicity is White.

^e^ADI: Area Deprivation Index.

^f^CCI: Charlson Comorbidity Index.

^g^SG: sleeve gastrectomy.

^h^The reference group for operation type is Roux-en-Y gastric bypass.

After controlling for other factors, a significantly lower expected %BMIR was observed in Black patients (β=–2.64, 95% CI –3.28 to –1.99; *P*<.001) compared with White patients (Table 2). Such difference was not significant for patients who were self-identified as other races or ethnicities (β=–1.55, 95% CI –3.24 to 0.14; *P*=.07). In terms of age at operation, a 5-year increase was found to be significantly associated with a 0.28% lower expected %BMIR (β=–.28, 95% CI –0.39 to –0.16; *P*<.001). When evaluating comorbidities, our findings indicated that patients with a higher CCI score were associated with a significantly lower expected %BMIR (β=–.30, 95% CI –0.47 to –0.13; *P*<.001). In the context of surgery type, our results indicated the expected %BMIR among the patients who underwent SG was 2.97% lower than those who received RYGB (β=–2.97, 95% CI –3.48 to –2.47; *P*<.001). In addition, patients from areas with different ADI did not show significant differences in expected %BMIR (β=.00, 95% CI –0.01 to 0.02; *P*=.59).

Regarding specific portal functions, “My Record” and “Visit” functions, which enable patients to access their medications and test results, and manage their appointments, respectively, were the most frequently used (Table 1). Our analysis found that the higher engagement in almost every portal function was associated with a significantly lower expected %BMIR at 3-month follow-up and a significantly higher expected %BMIR at 6- and 12-month follow-up (except the “Resources” function; Table 3). Specifically, “Resources,” “Others,” and “Billing” functions showed the strongest associations at 3 months (β=–2.63, 95% CI –5.07 to –0.20, *P*=.03 for “Resources”; β=–1.24, 95% CI –1.84 to –0.64, *P*<.001 for “Others”; β=–1.02, 95% CI –1.37 to –0.66, *P*<.001 for “Billing”). “Others,” “Billing,” and “Messaging” functions showed the strongest positive association at 6 and 12 months (at 6 months: β=3.28, 95% CI 2.35-4.21, *P*<.001 for “Others”; β=1.77, 95% CI 1.24-2.30, *P*<.001 for “Billing”; β=1.38, 95% CI 1.02-1.75, *P*<.001 for “Messaging”; at 12 months: β=1.97, 95% CI 0.32-3.62, *P*=.01 for “Others”; β=1.11, 95% CI 0.21-2.02, *P*=.01 for “Billing”; β=.78, 95% CI 0.10-1.46, *P*=.02 for “Messaging”).

**Table 3:** Model results of the estimated associations between specific portal functions early engagement and the percentage of BMI reduction by follow-up time (ie, 3, 6, and 12 months).

Portal function	3 months			6 months			12 months	
	β (95% CI)	*P* value	β (95% CI)		*P* value	β (95% CI)		*P* value
						
Billing	–1.02 (–1.37 to –0.66)	<.001	1.77 (1.24 to 2.30)		<.001	1.11 (0.21 to 2.02)		.01
Medical tools	–0.39 (–0.55 to –0.23)	<.001	0.89 (0.57 to 1.20)		<.001	0.59 (0.10 to 1.08)		.01
Messaging	–0.35 (–0.60 to –0.10)	.005	1.38 (1.02 to 1.75)		<.001	0.78 (0.10 to 1.46)		.02
My record	–0.38 (–0.57 to –0.18)	<.001	1.14 (0.85 to 1.44)		<.001	0.76 (0.22 to 1.30)		.006
Resources	–2.63 (–5.07 to –0.20)	.03	2.23 (–2.36 to 6.82)		.34	2.36 (–5.69 to 10.41)		.56
Visits	–0.30 (–0.48 to –0.12)	.001	1.06 (0.77 to 1.35)		<.001	0.59 (0.03 to 1.14)		.03
Others	–1.24 (–1.84 to –0.64)	<.001	3.28 (2.35 to 4.21)		<.001	1.97 (0.32 to 3.62)		.01

The sensitivity analysis (Figure 1 and Table S3 in Multimedia Appendix 1) indicated that the significant association between early portal engagement and %BMIR at 3, 6, and 12 months persisted after adjusting for baseline BMI (β=–.36, 95% CI –0.55 to –0.18, *P*<.001 at 3 months; β=1.11, 95% CI 0.82-1.31, *P*<.001 at 6 months; and β=.78, 95% CI 0.24-1.31, *P*=.004 at 12 months). Portal engagement increased significantly during the COVID-19 pandemic, with median engagement rising from 44.0 (IQR 26.0-68.8) active days before the pandemic to 71 (IQR 50-97) days amid it at 12 months after surgery (Table S4 in Multimedia Appendix 1). The analysis confirmed that the positive association between portal engagement and %BMIR was consistent across the prepandemic and pandemic periods (Tables S5 and S6 in Multimedia Appendix 1). In the sensitivity analysis of incorporating the interaction terms between portal engagement at different stages and age, there was no sufficient statistical evidence that the associations between portal engagements and weight loss outcome would vary by age (Table S7 in Multimedia Appendix 1).

## Discussion

### Principal Findings

Bariatric surgery necessitates continued postoperative care, and previous studies have suggested that patient portals can enhance health awareness and therapy adherence [11,37,38]. Echoing findings from these studies, our retrospective longitudinal study reveals complex relationships between portal engagement and weight loss outcomes following bariatric surgery. Specifically, engagement within the first 3 months (early portal engagement) was associated with poorer weight loss outcomes at the 3-month follow-up but associated with better outcomes at 6 and 12 months. Similarly, engagement between 3 and 6 months (early midterm portal engagement) was linked to less favorable outcomes at the 6-month follow-up. These patterns suggest that simultaneous portal usage during the same stage as the follow-up may reflect greater patient needs or complications, potentially leading to increased interactions with health care providers and resources. Over time, this heightened engagement is likely to support more effective health management, thereby improving long-term outcomes. This insight underscores the dynamic and crucial role of patient portals in patient care, emphasizing the importance of maintained engagement for long-term success in weight management after bariatric surgery.

Further investigation into the specific functions of the portal revealed that the “Messaging” functions have one of the strongest positive associations with %BMIR at 6-month follow-up, suggesting these functions could be particularly beneficial to postoperative management. Notably, by the 6-month follow-up, patients engaging with the “Messaging” functions for every 100 active days saw an additional reduction in BMI by 13.8%. These findings align with a previous study, which indicates that portal may be a source of context-based educational materials for self-management [12]. While “Billing” and “other functions” showed positive associations with %BMIR, they frequently co-occurred with other functions. In particular, “Billing” and “other functions” occurred together with “Visit” functions in 92% and 90% active days, respectively, suggesting that their statistical significance may be an artifact of metric design rather than evidence of clinically meaningful postoperative patient engagement through the portal.

### Comparison With Previous Work

Previous studies have highlighted the potential of digital health engagement in obesity treatment. For example, 1 study found that individuals with access to a mobile weight loss intervention tool achieved significantly greater weight loss at 6 months after surgery compared with those without access to the tool [39]. Another randomized controlled study demonstrated that patients who received a digital intervention before bariatric surgery engaged in more physical activity at an 8-week postoperative follow-up compared with those receiving usual care, although the intervention did not significantly affect BMI [40]. In addition, a survey study has shown that online forums support patients by providing information and fostering motivation [41]. These findings are consistent with the results of our study, However, previous studies focused on different types of digital health engagement. Our study focused on the patient portal, which is integrated with EHRs and an inevitable method to deliver health service. Furthermore, we are the first longitudinal study to investigate the influence of engagement at different time stages on weight loss outcomes. Suggesting that digital tools could serve as a valuable complement to standard perioperative and postoperative care in bariatric surgery.

Similar to previous bariatric surgery studies, loss to follow-ups remains a significant challenge [40-43]. In our study, follow-up rates were 95.1%, 76.7%, and 66.9% at 3, 6, and 12 months, respectively. Potential reasons for this loss included patients with fewer symptoms opting out of later follow-up appointments at the surgery center, unsuccessful weight loss (<50% excess weight loss), changes in their residence and insurance status, and so on [42,43]. This decline in follow-up rates from 6 months to 12 months may reduce the power to capture the association between portal engagement and BMI reduction and may cause a potential selection bias at 12 months. Since the reasons for patients’ loss to follow-up visits were unknown, it may potentially cause an overestimation or underestimation of the benefits of patient portal engagement.

In addition to patient portal engagement, we observed that younger patients tended to achieve better postoperative outcomes (Table 2). This could be attributed to faster metabolic rates in younger individuals [44]. In addition, patient’s digital access and technical skills may also vary by age [33]. However, age cannot be used as a proxy for these since age’s relationship with the weight loss outcome is complex. Our findings align with previous studies that reported racial differences in weight loss after bariatric surgery, noting that self-identified Black patients experienced less weight loss compared with their White counterparts; however, the underlying causative reasons remain unclear [45]. We also found that patients with multiple comorbidities exhibited less weight loss, highlighting the need for tailored postoperative care for patients with more complex health conditions. Our analysis revealed that patients who underwent RYGB achieved more weight loss than those who had SG, consistent with existing literature [8,46]. The duration since surgery was positively associated with BMI reduction. It highlighted the importance of considering the timeline in post–bariatric surgery outcomes analysis. While these observations were significant, they were not the primary focus of this work and warrant further investigation.

### Clinical Significance

Our research provides in-depth, longitudinal analysis of patient portal use in bariatric surgery, highlighting its benefits in the management of postoperative weight loss resources and the communication between clinicians and patients. However, more needs to be done to familiarize both patients and health care professionals with portal usage. Encouragement from health care providers and user-friendly designs from vendors are essential [47-49]. Nonetheless, the increasing use of portals may lead to clinician burnout [50,51], underscoring the need for strategies that balance efficient digital interaction management with health care team well-being. Achieving this equilibrium is crucial for maximizing patient satisfaction and the effective use of digital health tools.

### Future Directions

Building on the insights from our data-driven study, the next steps should focus on hypothesis-driven research to delve deeper into the nuances of patient portal engagement and its impact on postoperative weight management in patients after bariatric surgery. Recommended future studies include conducting randomized controlled trials to test the effectiveness of targeted portal interventions at different postoperative stages, exploring the reasons behind patient engagement through qualitative methods, and incorporating demographic variables like age to personalize portal use. In addition, longitudinal randomized controlled trials could rigorously test the causality between portal use and weight loss, while expanding research to diverse populations could validate the generalizability of the findings. Optimizing portal features based on the most impactful functions and evaluating collaborative care models that integrate real-time, provider-patient interactions through portals would also be beneficial. These steps would refine understanding and enhance the efficacy of patient portals in supporting long-term weight management success post bariatric surgery.

### Strengths and Limitations

Several limitations in this study are worth acknowledging. First, we would like to clarify that our research was not to establish causality. Our study was a data-driven, retrospective study designed to measure the “association” between postoperative patient portal usage and %BMIR at 3, 6, and 12 months after surgery. The insights gleaned from our findings are intended to inform the design of future clinical trials. Second, this study was conducted at a single academic medical center. The generalizability of the findings needs to be confirmed in other large surgical weight loss programs. Third, we acknowledged the need for better data collection during presurgery screening and postoperative follow-up visits. While we adjusted for a range of common factors associated with bariatric surgery outcomes, there could be confounding effects from other factors not within our data access, such as individual-level socioeconomic status, lifestyle factors such as diet and physical activity levels, health literacy, digital literacy, digital access, reasons for loss to follow-up visits, and engagement with other health monitoring apps or other forms of interventions. Due to the limitation of our retrospective study, these variables had low coverage in our cohort. Fourth, we observed a data imbalance in the proportion of Black patients between the surgery groups. Although we adjusted for the surgery type and race and ethnicity as covariates, the imbalance may influence the observed association between race and ethnicity and %BMIR. To address this potential concern, one approach would be to explore the association within each surgery type separately. Finally, the direct effects of specific clinic-oriented activities conducted through the portal—such as medication refill requests or appointment scheduling—on improved weight loss outcomes have yet to be determined. In this study, these actions were not investigated in detail due to the smaller sample sizes they represent, which could result in a lack of statistical power and unstable estimates. Moving forward, our research aims to identify and scrutinize these pivotal actions more closely and assess their associations with post–bariatric surgery outcomes.

### Conclusions

In this retrospective study, we analyzed longitudinal data from the VUMC bariatric surgery registry alongside patient portal use records. Our findings indicate that higher early portal engagement was associated with less favorable weight loss immediately after surgery but associated with improved outcomes at 6 to 12 months. The portal engagement metric and longitudinal analysis framework developed in this study hold promise for application in other chronic conditions requiring long-term management. Future research should aim to include additional postoperative factors such as dietary intake and comorbidity improvements to provide a more comprehensive understanding of the long-term outcomes following bariatric surgery.

## Appendix

Multimedia Appendix 1 Supplementary materials.

## Abbreviations

%BMIR: percentage of BMI reduction
ADI: Area Deprivation Index
CCI: Charlson Comorbidity Index
EHR: electronic health record
IRB: institutional review board
MBSAQIB: Metabolic and Bariatric Surgery Accreditation and Quality Improvement Program
MHAV: My Health at Vanderbilt
RESI: robust effect size index
RYGB: Roux-en-Y gastric bypass
SG: sleeve gastrectomy
STROBE: Strengthening the Reporting of Observational Studies in Epidemiology
VUMC: Vanderbilt University Medical Center
